# Gastrointestinal Manifestations and Associated Health Outcomes of COVID-19: A Brazilian Experience From the Largest South American Public Hospital

**DOI:** 10.6061/clinics/2020/e2271

**Published:** 2020-10-21

**Authors:** Diogo Turiani Hourneaux de Moura, Igor Mendonça Proença, Thomas R. McCarty, Vitor Massaro Takamatsu Sagae, Igor Braga Ribeiro, Guilherme Henrique Peixoto de Oliveira, Gabriel Mayo Vieira de Souza, Bruno Salomão Hirsch, Maria Vitória Cury Vieira Scatimburgo, Christopher C. Thompson, Flair José Carrilho, Ivan Cecconello, Eduardo Guimarães Hourneaux de Moura

**Affiliations:** I Hospital das Clinicas (HCFMUSP), Faculdade de Medicina, Universidade de Sao Paulo, SP, Brazil; II Brigham and Women’s Hospital - Harvard Medical School, Boston 02115, MA, United States

**Keywords:** COVID-19, SARS-CoV-2, Gastrointestinal Symptoms, Prevalence, Pandemic

## Abstract

**OBJECTIVES:**

Brazil has rapidly developed the second-highest number of COVID-19 cases in the world. As such, proper symptom identification, including gastrointestinal manifestations, and relationship to health outcomes remains key. We aimed to assess the prevalence and impact of gastrointestinal symptoms associated with COVID-19 in a large quaternary referral center in South America.

**METHODS:**

This was a single-center cohort study in a COVID-19 specific hospital in São Paulo, Brazil. Consecutive adult patients with laboratory confirmed SARS-CoV-2 were included. Baseline patient history, presenting symptoms, laboratory results, and clinically relevant outcomes were recorded. Regression analyses were performed to determine significant predictors of the gastrointestinal manifestations of COVID-19 and hospitalization outcomes.

**RESULTS:**

Four-hundred patients with COVID-19 were included. Of these, 33.25% of patients reported ≥1 gastrointestinal symptom. Diarrhea was the most common gastrointestinal symptom (17.25%). Patients with gastrointestinal symptoms had higher rates of concomitant constitutional symptoms, notably fatigue and myalgia (*p*<0.05). Gastrointestinal symptoms were also more prevalent among patients on chronic immunosuppressants, ACE/ARB medications, and patient with chronic kidney disease (*p*<0.05). Laboratory results, length of hospitalization, ICU admission, ICU length of stay, need for mechanical ventilation, vasopressor support, and in-hospital mortality did not differ based upon gastrointestinal symptoms (*p*>0.05). Regression analyses showed older age [OR 1.04 (95% CI, 1.02-1.06)], male gender [OR 1.94 (95% CI, 1.12-3.36)], and immunosuppression [OR 2.60 (95% CI, 1.20-5.63)], were associated with increased mortality.

**CONCLUSION:**

Based upon this Brazilian study, gastrointestinal manifestations of COVID-19 are common but do not appear to impact clinically relevant hospitalization outcomes including the need for ICU admission, mechanical ventilation, or mortality.

## INTRODUCTION

The new coronavirus infection, as known as Severe Acute Respiratory Syndrome Coronavirus 2 (SARS-CoV-2), was first reported in Wuhan, China in late 2019 (1). The virus has quickly spread across the world, becoming a pandemic as declared by the World Health Organization in March 2020 (2). As of July 2020, more than 10 million confirmed cases of Coronavirus Disease (COVID-19) across five continents and over 500 thousand deaths have been reported (3). While respiratory symptoms are the main presentation of COVID-19, such as dry cough and dyspnea gastrointestinal manifestations have also been reported (4,5). As the number of cases has increased, so too has our knowledge grown about various symptoms associated with the SARS-CoV-2 infection.

SARS-CoV-2 is known to affect host cells via the angiotensin-converting enzyme receptor (ACE2), which in addition to being highly expressed in pulmonary AT2 cells, are also found in the gastrointestinal system such as cells in the esophagus, pancreas, hepatobiliary tract, small bowel, and colon - indicating that in addition to the respiratory system, the gastrointestinal system is a possible means of infection by COVID-19 (6). Several studies report patients with COVID-19 presenting with concomitant or isolated gastrointestinal symptoms; however, there remains a paucity of data from the continent of South America (7-9). Although the United States has become the new epicenter of COVID-19 with 2.84 million confirmed cases, Brazil has become an emerging hot bed of SARS-CoV-2 infection with 1.54 million cases reported to date - approximately 13.5% of all confirmed cases (10). As disease prevalence, presenting symptoms, and outcomes have varied from reports in China to those in the United States and Europe, examination of COVID-19 specific characteristics is highly relevant for the population of Brazil and South America. Therefore, the primary aim of this study was to assess the prevalence of gastrointestinal symptoms associated with SARS-CoV-2 infection and examine gastrointestinal-specific health outcomes in a quaternary referral hospital exclusively treating COVID-19 patients in São Paulo, Brazil (11).

## MATERIAL AND METHODS

### Study Design and Patient Selection

This is a single-center cohort study in a quaternary hospital specifically treating patients with COVID-19 in São Paulo, Brazil. Out the outset of the pandemic, this quaternary university referral center instituted a protocol to exclusively care for COVID-19 patients. This hospital and institution is comprised of the largest public hospital in South America (11). The hospital is designed to care for patients presenting with moderate and severe disease and receives patient referrals following local government protocol. A total of 400 consecutive, adult patients underwent complete hospitalization from May 1 to June 20, 2020, and were included in this analysis. All patients were followed to hospital discharge or death. Inclusion criteria for hospitalization and study enrollment comprised of only patients with laboratory confirmed SARS-CoV-2 via polymerase-chain reaction (PCR). Patients with suspicion for COVID-19 based upon symptoms alone or imaging with computed tomography (CT) without PCR confirmation were excluded from this analysis.

### Data Items

Demographic patient data (age and gender), and symptoms at the time of presentation were recorded. Symptoms were stratified initially as general symptoms (fever, fatigue, myalgia, chills, arthralgia, or diaphoresis), respiratory symptoms (cough, productive cough, dyspnea, pharyngitis, or rhinorrhea), gastrointestinal symptoms (diarrhea, nausea, anorexia, vomiting, abdominal pain, dysphagia, weight loss, gastrointestinal bleeding, or constipation), as well as other non-specific symptoms including anosmia/ageusia. Additional data abstracted through manual chart review included past medical history including pre-existing comorbid medical conditions, chronic use of angiotensin converting enzyme-inhibitor (ACE-I) or angiotension receptor blocker (ARB), chronic use of immunosuppressant medications, clinically relevant laboratory data at time of presentation, and relevant hospitalization characteristics (hospitalization days, intensive care unit [ICU], admission, need for mechanical ventilation, need for vasopressors support, and mortality). All data was abstracted manually from electronic medical records using a structured abstraction tool.

### Outcomes

The primary outcome was the to evaluate the impact of gastrointestinal symptoms among COVID-19 patients and clinically relevant health outcomes including need for ICU stay, mechanical ventilation, and in-hospital mortality. Secondary analyzes included assessment of prevalence of any gastrointestinal symptoms among patients hospitalized with COVID-19 at initial presentation, associations between gastrointestinal symptoms and other clinical manifestations, comorbidities, and laboratory results.

### Ethical Concerns

This study was approved by the Research Ethics Committee of Hospital das Clínicas - University of São Paulo Medical School (HC-FMUSP).

### Statistical Analyses

Baseline patient characteristics, COVID-19 manifestations, laboratory data, as well as hospitalization outcomes were summarized as means ± standard deviation for continuous data and frequencies and proportions for categorical data. Continuous data were compared using the two-sample t-test or Wilcoxon rank-sum test and categorical data were compared using the Chi-square or Fisher’s exact test, as appropriate (12). Multivariable analyses were performed using logistic regression. Logistic regression analyses were conducted to determine significant predictors of the gastrointestinal manifestations of COVID-19 and hospitalization outcomes and were reported as standardized β coefficients as well as odds ratio (OR) with corresponding 95% confidence intervals (CIs). With regard to gastrointestinal symptoms, a regression analysis was performed based upon the 3 most common manifestations while key hospitalization outcomes included need for ICU admission, need for mechanical ventilation, or in-hospital mortality. Variables for regression analyses included age, gender, obesity, chronic use of ACE-I or ARB and use of immunosuppressant medications, with additional variables determined based upon significant findings on univariable analyses. Statistical significance was defined as a two-tailed *p* value <0.05. Statistical analyses were performed using the Stata 15.0 software package (Stata Corp LP, College Station, TX).

## RESULTS

### Patients Characteristics

This study included 400 patients with COVID-19 with laboratory confirmed SARS-CoV-2 (COVID-19). Of these patients included in this analysis, 56.25% (n=225) were male, with an average age of 56.4 ± 16.07 years. The most frequent comorbid medical conditions among this population included hypertension (54.64%; n=218), diabetes mellitus (35.93%; n=143), and obesity (21.55%; n=86). A complete breakdown of demographic information and comorbidities is summarized in Table 1.

### Gastrointestinal symptoms at presentation

A total of 133 (33.25%) patients reported at least one gastrointestinal symptom at the time of presentation, with diarrhea (17.25%) being the most prevalent. Other common gastrointestinal manifestations included nausea (13.75%) and anorexia (11.5%), followed by vomiting and abdominal pain observed 7.50% and 6.0% of individuals, respectively. A complete list of gastrointestinal symptoms at the time of presentation among patients is summarized in Table 2.

### Correlation between gastrointestinal symptoms and other manifestations

Patients with *gastrointestinal* symptoms had higher rates of constitutional symptoms, including fatigue (59.40% vs 44.57%; *p*=0.0051) and myalgia (42.11% vs 29.96%; p=0.0157). In addition, ageusia was a more common among these patients (22.56% vs 11.24%; *p*=0.0027). The occurrence of respiratory symptoms did not predominate in a group with or without gastrointestinal symptoms (90.98% vs 93.26%; *p*=0.4158). Further breakdown of constitutional, respiratory, and other symptoms as stratified by the presence of absence of gastrointestinal manifestations is described in Table 3.

### Correlation between gastrointestinal symptoms and medical history

Among included patients, 117 (29.62%) were chronic users of an ACE-I or ARB medication. Forty-five (11.28%) patients were prescribed and taking immunosuppressive medication. Regarding the use of specific medications, there was an association between gastrointestinal symptoms and the use of ACE-I or ARBs (38.46% vs 25.28%; *p*=0.0069). Patients using immunosuppressants also had a higher rate of gastrointestinal symptoms (15.79% vs 9.02%; *p*=0.0441). Patients with chronic kidney disease had a higher prevalence of gastrointestinal symptoms (22.56% vs 12.41%; *p*=0.0087), while patient with previous cerebrovascular accident had lower prevalence (0.75% vs 4.89%; *p*=0.034). Other comorbidities were not significantly different between groups (Table 1).

### Correlation between gastrointestinal symptoms and laboratory data

Although some laboratory tests were altered such as lymphocytes, C-reactive protein, and lactate dehydrogenase, there was no statistically significant difference between patients with and without gastrointestinal symptoms at time of presentation. Hematological parameters as well as other inflammatory markers were also similar between groups (Table 1).

### Correlation between gastrointestinal symptoms and clinical outcomes

Main outcomes related to COVID-19 such as length of hospitalization, need for ICU admission, ICU length of stay, need of mechanical ventilation, and need for vasopressor support did not differ between patients with or without gastrointestinal symptoms (Table 1). There was also no significant difference for in-hospital mortality rates between the two groups (21.05% vs 22.85%, *p*=0.6896).

### Regression analyses for gastrointestinal symptoms and clinical outcomes

A multivariable logistic regression analysis was then performed for the 3 most common gastrointestinal manifestations to determine their impact on clinically relevant health outcomes (Table 4). After controlling for confounders, diarrhea was more common among patients with a history of ACE-I or ARB use [OR 1.87 (95% CI 1.04 to 3.36); *p*=0.036], those on immunosuppressant medications [OR 2.54 (95% CI 1.19 to 5.41); *p*=0.016], and presenting symptoms of fever [OR 3.47 (95% CI 1.66 to 7.27); *p*=0.001]. With regard to anorexia, older age [OR 0.21 (95% CI 1.04 to 1.05); *p*=0.021] and loss of taste or smell were predictors [OR 2.83 (95% CI 1.32 to 6.05); *p*=0.007] while ACE-I or ARB use [OR 2.05 (95% CI 1.07 to 3.95); *p*=0.031] or ageusia or anosmia were predictors for nausea [OR 2.12 (95% CI 1.04 to 4.32); *p*=0.039].

Among included patients, gastrointestinal symptoms did not appear to be influence hospitalization outcomes after controlling for other variables (Table 4). Significant predictors of admission to the ICU included male gender [OR 1.60 (95% CI 1.04 to 2.46); *p*=0.032], obesity [OR 2.18 (95% CI 1.30 to 3.67); *p*=0.003] and presence of fatigue [OR 1.64 (95% CI 1.03 to 2.60); *p*=0.036]. Myalgia and ageusia or anosmia appeared to be protective factors in relation to admission to ICU [OR 0.52 (95% CI 0.32 to 0.85); *p*=0.009] and [OR 0.38 (95% CI 0.20 to 0.71); *p*=0.003, respectively].

The need for mechanical ventilation in patients with gastrointestinal symptoms was related to male gender [OR 1.63 (95% CI 1.05 to 2.55); *p*=0.030] and obesity [OR 2.33 (95% CI 1.39 to 3.95); *p*=0.001]. There was also a negative correlation between mechanical ventilation and use of ACE-I or ARB medications [OR 0.58 (95% CI 0.36 to 0.96); *p*=0.033], the presence of myalgia [OR 0.56 (95% CI 0.33 to 0.94); *p*=0.027] and ageusia or anosmia [OR 0.27 (95% CI 0.13 to 0.57); *p*=0.001].

Mortality was associated with older age [OR 1.04 (95% CI 1.02 to 1.06); *p*<0.001], male gender [OR 1.94 (95% CI 1.12 to 3.36); *p*=0.018] and use of immunosuppressants [OR 2.60 (95% CI 1.20 to 5.63); *p*=0.016]. There was a negative relationship between mortality in patients with gastrointestinal symptoms and use of ACE-I or ARB [OR 0.54 (95% CI 0.30 to 0.97); *p*=0.041].

## DISCUSSION

In this single-center study of quaternary referral care center in São Paulo, Brazil, we found approximately one-third of the patients (33.25%) presented with at least one gastrointestinal symptom. Diarrhea (17.25%), nausea (13.75%), and anorexia (11.5%) were the most prevalent symptoms. However, the presence of gastrointestinal symptoms did not impact need for ICU admission, mechanical ventilation, or all-cause inpatient mortality. To date, this is the largest Brazilian study to report the prevalence of gastrointestinal symptoms in a cohort of COVID-19 hospitalized patients and evaluate the impact of gastrointestinal manifestations among key health outcomes.

Interestingly, the prevalence of gastrointestinal symptoms (33.25%), most notably diarrhea, has been highly variable worldwide - with our results approximately 3-times higher than previous meta-analyses have demonstrated (10% to 13%)(5,13,14). However, a recent multi-center study in the United States, not included in those meta-analyses, has reported a prevalence as high as 61.3%(7). A plausible explanation may be that hospitalized patients have a more systemic disease and thus, would have a higher prevalence of gastrointestinal symptoms. Additionally, the 10% to 13% prevalence reported in these meta-analyses is based upon inpatient and outpatient populations. While this may vary on a global scale, the referral natural of this hospital caring for moderate-to-severe disease may result in a greater prevalence of gastrointestinal manifestations. This is supported in this study by the observation that patients with gastrointestinal symptoms had more general symptoms than patients without gastrointestinal. Anosmia and/or ageusia also had a higher prevalence among patients with gastrointestinal symptoms, similar to an association previously highlighted in a United States study (7).

While baseline demographic and laboratory data were not different between patients with or without gastrointestinal symptoms, history of a cerebrovascular accident was less prevalent among patients presented with gastrointestinal symptoms (*p*=0.0344). This is a new negative association found in this study though difficult to truly explain - and may be difficult to determine given the few patients with gastrointestinal manifestations. On the other hand, chronic kidney disease was more prevalent among patients with GI symptoms (*p*=0.0087), which could potentially be explained by chronic use of ACE-I/ARB among those patients - also more common among patients with gastrointestinal manifestations. It is known that SARS-CoV-2 enters host cells via cell receptor ACE2 (6). ACE2 is highly expressed in the respiratory tract cells but also in the gastrointestinal tract cells (6). That may explain the high prevalence of gastrointestinal symptoms among patients with COVID-19 and the association with ACE-I/ARB chronic use - usually taken by patients with chronic kidney disease , which could upregulate natural receptors for the virus on those cells (15,16).

In this study, after a multivariable logistic regression, diarrhea was associated with use of ACE-I/ARB [OR 1.87 (95% CI 1.04 to 3.36); *p*=0.036]. However, the role of ACE-I/ARB and COVID-19 symptoms remains controversial. Initial concerns were raised about ACE2 upregulation in patients in chronic use of ACE-I/ARB and its impact on severity and mortality of COVID-19 (17). At least one study supposed a higher mortality among these patients (18). Two meta-analyses have reached different results: one demonstrating no association between ACE-I/ARB chronic use with disease severity (19) while the other one showed a possible protective effect with lower mortality among ACE-I/ARB users (20). Our study reported that chronic use of ACE-I/ARB is a protective factor for mechanical ventilation and associated with a lower mortality. A Randomized Clinical Trial comparing suspending or continuing use of ACE-I/ARB in chronic users is currently under way and should aid in understanding the role of those drugs among COVID-19 patients (21).

The main outcomes related to COVID-19 infection such as hospitalization days, ICU admission, ICU days, endotracheal intubation, and need of vasopressor support did not differ between patients with or without gastrointestinal symptoms. Although gastrointestinal symptoms are frequent and may be associated with specific constitutional symptoms, this finding suggests that gastrointestinal symptoms are not associated to severity of disease nor worse outcomes - a finding consistent with other literature (7,22). Two meta-analyses found relation between abdominal pain and severe disease, but this did not significantly impact mortality. Severe disease was reported with high heterogeneity between those studies, including oxygen saturation parameters, pulmonary involvement on image exams, and ICU admission, which may lead to imprecision on analyses (13,14).

On multivariable logistic regression, myalgia and anosmia/ageusia were associated with a decrease in need for ICU admission and mechanical ventilation. Myalgia was even associated with lower mortality among hospitalized patients. Classically, myalgia and anosmia/ageusia are reported as early symptoms in COVID-19 (23,24) which may translate in our study to mean earlier diagnosis and possibly result in an improved prognosis. Although myalgia is commonly associated with generalized inflammation and cytokine response, a meta-analysis showed that myalgia is not relate with severity or mortality (24). With regards of anosmia/ageusia, a systematic review that included 42 studies showed an inverse relation with severity and hospitalization, suggesting that anosmia/ageusia are more frequently associated with mild-to-moderate COVID-19 (25).

Among patients with gastrointestinal symptoms, obesity and male gender were associated with a higher rate of ICU admission and need for mechanical ventilation. Studies, including one meta-analysis, have shown obesity and male gender are associated with more severe disease and poorer outcomes in general population (25,26). Fatigue was also an independent factor associated with more ICU admission among patients (25). Our study demonstrated that fatigue was also associated with more severe disease (i.e., ICU admission). This is an important finding that should prompt attention to the possibility for more severe disease or a prolonged hospital course should patients report fatigue on presentation. With regards to increased mortality, older age, male gender, and immunosuppressed patients were predictors - similar to what is known in the general population and for a variety of other illnesses (25,26).

Despite this is the largest single-center Western study related to patients with COVID-19 and gastrointestinal symptoms, our study is not without limitations. First, the retrospective study design and manual chart review may introduce the possibility of bias - including possible missed or incomplete data, poor medical documentation, and potential for under or over appreciating symptoms at time of hospital presentation. Furthermore, the referral based nature of the hospital may introduce selection bias and inclusion of only hospitalized patients. Yet despite these limitations, our study possesses several strengths. Notably, this study provides important insights regarding COVID-19 from the largest hospital in Brazil, a nation with the second most confirmed cases in the world. As the rate continues to skyrocket, increasing faster than that of the United States, examination of this unique population may prove exceedingly important to determine next steps in identification and understanding of the disease in a South American population.

## CONCLUSION

Gastrointestinal symptoms are prevalent among patients with COVID-19, with diarrhea being the most common manifestation. Patients in chronic use of ACE-I/ARB are more likely to present with gastrointestinal symptoms, as well as the concomitant presentation of myalgia or anosmia/ageusia with gastrointestinal symptoms. Based upon this analysis, gastrointestinal symptoms do not appear to impact key COVID-19 associated hospitalization outcomes including the need for ICU admission, mechanical ventilation, or all-cause inpatient mortality. Older age, male gender, and immunosuppressed patients were the only conditions associated with higher mortality.

## Conflict of Interest

Dr. Christopher C. Thompson reports fee as a consultant for Boston Scientific and Medtronic; fees as consultant and institutional grants from USGE Medical, Olympus, and Apollo Endosurgery. Dr. Eduardo Guimarães Hourneaux de Moura reports personal fees from Boston Scientific, personal fees from Olympus, outside the submitted work.

The others authors reported no potential conflict of interest.

## AUTHOR CONTRIBUTIONS

de Moura DTH participate in the study concept and design, manuscript preparation, critical revisions. Proença IM participate in the acquisition of data, manuscript and data preparation, critical revisions. Sagae VMT participate in the study concept and design, data acquisition and interpretation, critical revisions. McCarty TR participate in the statistical analyses, data interpretation, critical revisions. Ribeiro IB participate in the acquisition of data, manuscript preparation, data interpretation, critical revisions. Hirsch BS, De Oliveira GHP, De Souza GMV and Scatimburgo MVCV participated in the acquisition of data, statistical analyses, data interpretation. Thompson CC participate in the study concept and design, critical revisions. Carrilho FJ and Cecconell I participated in the critical revisions. de Moura, EGH participate in the study concept and design, data interpretation, critical revisions.

All authors approve of the final version of the manuscript.

## Figures and Tables

**Table 1:** Baseline Clinical Characteristics, Comorbidities, Social Factors, Medications, and Laboratory Data on Admission, and Outcomes of COVID-19 Hospitalizations.

	All Patients (n=400)	GI Symptoms (n=133)	No GI Symptoms (n=267)	*P* Value
Baseline Characteristics				
Age	56.40 (16.07)	57.52 (15.95)	55.83 (16.12)	0.3208
Male Gender	225 (56.25)	79 (59.40)	146 (54.68)	0.3716
Comorbid Conditions				
Obesity	86 (21.55)	24 (18.05)	62 (23.31)	0.2291
Coronary Artery Disease	43 (10.78)	12 (9.02)	31 (11.65)	0.4255
Heart Failure	39 (9.77)	8 (6.02)	31 (11.65)	0.0741
Cardiac Arrythmia	29 (9.77)	10 (7.52)	19 (7.14)	0.8919
Hypertension	218 (54.64)	76 (57.14)	143 (53.38)	0.4783
Dyslipidemia	36 (9.05)	12 (9.02)	24 (9.06)	0.9911
Diabetes	143 (35.93)	45 (34.09)	98 (36.84)	0.5913
Cerebrovascular Accident	14 (3.51)	1 (0.75)	13 (4.89)	0.0344
Pulmonary Disease	32 (8.02)	10 (7.52)	22 (8.27)	0.795
Chronic Kidney Disease	62 (15.79)	30 (22.56)	32 (12.41)	0.0087
Thyroid Disease	36 (9.02)	17 (12.78)	19 (7.14)	0.0641
Malignancy	49 (12.31)	15 (11.45)	34 (12.73)	0.759
Social Factors				
Active Tobacco Use	16 (4.01)	5 (3.76)	11 (4.14)	0.8573
Former Tobacco Use	71 (17.88)	30 (22.56)	41 (15.53)	0.0851
Alcohol Use Disorder	19 (4.76)	6 (4.51)	13 (4.89)	0.8684
Medications				
Immunosuppressants	45 (11.28)	21 (15.79)	24 (9.02)	0.0441
ACE-I or ARB	117 (29.62)	50 (38.46)	67 (25.28)	0.0069
Laboratory Data				
Hemoglobin in g/dL	11.87 (2.20)	11.96 (2.12)	11.83 (2.24)	0.5792
Leukocytes in in /microL	9288.38 (13721.94)	8748.03 (8330.11)	9556.51 (15634.12)	0.5821
Lymphocytes in in /microL	1620.31 (9702.31)	1073.87 (546.75)	1893.52 (11874.71)	0.4305
Platelets x1000/microL	228 (122)	223 (106)	230 (129)	0.5816
ALT in U/L	46.94 (49.89)	49.06 (51.16)	45.79 (49.26)	0.5525
AST in U/L	50.92 (48.91)	56.01 (64.77)	48.18 (37.55)	0.1463
Total Bilirubin in mg/dL	0.56 (1.00)	0.48 (0.46)	0.61 (1.19)	0.2858
Direct Bilirubin in mg/dL	0.44 (0.98)	0.35 (0.44)	0.48 (1.17)	0.294
Alkaline Phosphatase in U/L	120.55 (130.45)	106.21 (78.15)	128.38 (151.27)	0.247
GGT in U/L	195.91 (251.16)	175.73 (207.03)	206.68 (271.93)	0.4032
Amylase in U/L	76.77 (51.92)	77.71 (45.71)	75.94 (57.53)	0.8961
INR	1.17 (0.79)	1.09 (0.24)	1.20 (0.94)	0.2579
D-Dimer in ng/mL	4648.45 (12025.48)	3954.72 (10423.51)	5027.44 (12823.68)	0.4367
NT-pro-BNP in pg/mL	4145.42 (11212.44)	3425.63 (8471.85)	4552.58 (12522.54)	0.5495
Lactic acid in mg/dL	15.35 (18.75)	13.56 (6.38)	16.28 (22.58)	0.2614
LDH in U/L	416.28 (238.18)	397.13 (232.45)	426.83 (241.17)	0.2753
CRP in mg/dL	141.87 (110.53)	130.60 (98.64)	147.60 (115.88)	0.1601
Hospitalization Characteristics				
Length of Hospital Stay in days	14.15 (10.71)	13.77 (9.66)	14.34 (11.21)	0.6153
ICU Admission	201 (50.25)	62 (46.62)	139 (52.06)	0.3062
Length of ICU Stay in days	12.16 (8.52)	12.02 (9.13)	12.22 (8.28)	0.8732
Endotracheal Intubation	161 (40.25)	48 (36.09)	113 (42.32)	0.2322
Vasopressor Support	142 (35.50)	42 (31.58)	100 (37.45)	0.2485
Mortality	89 (22.25)	28 (21.05)	61 (22.85)	0.6896

**Table 2:** Prevalence of Gastrointestinal Symptoms at Time of Presentation Among Hospitalized Patients with COVID-19.

Prevalence of Gastroenterology (GI) Symptoms	
≥1 Symptom	133 (33.25)
Diarrhea	69 (17.25)
Nausea	55 (13.75)
Anorexia	46 (11.5)
Vomiting	30 (7.50)
Abdominal Pain	24 (6.00)
Dysphagia	3 (0.75)
Weight Loss	2 (0.50)
GI Bleeding	4 (1.00)
Constipation	2 (0.50)

**Table 3:** Constitutional, Respiratory, and Other Symptoms at Time of Presentation Among Hospitalized Patients with COVID-19.

	All Patients	GI Symptoms (n=133)	No GI Symptoms (n=267)	*P* Value
General Symptoms				
≥1 Symptom	275 (82.25)	117 (87.97)	212 (79.40)	0.0347
Fever	69.5	99 (74.43)	179 (67.04)	0.1308
Fatigue	49.5	79 (59.40)	119 (44.57)	0.0051
Myalgia	34	56 (42.11)	80 (29.96)	0.0157
Chills	4.25	7 (5.26)	10 (3.75)	0.4796
Arthralgias	5.25	9 (6.77)	12 (4.49)	0.3383
Diaphoresis	1.25	3 (2.26)	2 (0.75)	0.204
Respiratory Symptoms				
≥1 Symptom	370 (92.50)	121 (90.98)	349 (93.26)	0.4158
Cough	291 (72.75)	103 (77.44)	188 (70.41)	0.1374
Productive Cough	22 (8.25)	10 (7.52)	23 (8.61)	0.7084
Dyspnea	332 (82.96)	107 (81.61)	224 (83.90)	0.4799
Pharyngitis	17 (4.25)	4 (3.01)	13 (4.87)	0.3859
Rhinorrhea	40 (10.00)	9 (6.77)	31 (11.61)	0.1288
Other Symptoms				
Ageusia/Anosmia	68 (17.00)	33 (24.81)	35 (13.11)	0.0033
Ageusia	60 (15.00)	30 (22.56)	30 (11.24)	0.0027
Anosmia	46 (11.50)	20 (15.04)	26 (9.74)	0.1181

**Table 4:** Multivariable Logistic Regression Model for Gastrointestinal Symptoms.

Multivariable Regression Model for Diarrhea	Odds Ratio (95% CI)	*P* Value	Multivariable Regression Model for ICU Admission	Odds Ratio (95% CI)	*P* Value
Age	1.00 (0.98 to 1.02)	0.856	Age	1.00 (0.98 to 1.01)	0.684
Gender	1.21 (0.68 to 2.13)	0.519	Gender	1.60 (1.04 to 2.46)	0.032
Obesity	0.71 (0.35 to 1.44)	0.345	Obesity	2.18 (1.30 to 3.67)	0.003
ACE-I or ARB	1.87 (1.04 to 3.36)	0.036	ACE-I or ARB	0.78 (0.49 to 1.25)	0.305
Immunosuppressants	2.54 (1.19 to 5.41)	0.016	Immunosuppressants	1.19 (0.61 to 2.31)	0.605
Tobacco Use	1.90 (0.48 to 7.42)	0.358	≥1 GI Symptom	0.90 (0.57 to 1.41)	0.631
Alcohol Use	0.42 (0.09 to 2.01)	0.28	Myalgia	0.52 (0.32 to 0.85)	0.009
Fever	3.47 (1.66 to 7.27)	0.001	Fatigue	1.64 (1.03 to 2.60)	0.036
Ageusia or Anosmia	1.45 (0.74 to 2.85)	0.28	Ageusia or Anosmia	0.38 (0.20 to 0.71)	0.003

Multivariable Regression Model for Diarrhea	Odds Ratio (95% CI)	*P* Value	Multivariable Regression Model for ICU Admission	Odds Ratio (95% CI)	*P* Value
Age	1.03 (1.01 to 1.05)	0.021	Age	1.01 (0.99 to 1.02)	0.28
Gender	1.08 (0.54 to 2.17)	0.82	Gender	1.63 (1.05 to 2.55)	0.03
Obesity	1.06 (0.47 to 2.39)	0.879	Obesity	2.33 (1.39 to 3.95)	0.001
ACE-I or ARB	1.45 (0.72 to 2.91)	0.294	ACE-I or ARB	0.58 (0.36 to 0.96)	0.033
Immunosuppressants	1.11 (0.39 to 3.13)	0.844	Immunosuppressants	1.48 (0.74 to 2.92)	0.261
Tobacco Use	0.65 (0.08 to 5.39)	0.69	≥1 GI Symptom	0.87 (0.55 to 1.39)	0.567
Alcohol Use	1.97 (0.50 to 7.82)	0.336	Myalgia	0.56 (0.33 to 0.94)	0.027
Fever	1.78 (0.80 to 3.98)	0.16	Fatigue	1.30 (0.81 to 2.07)	0.275
Ageusia or Anosmia	2.83 (1.32 to 6.05)	0.007	Ageusia or Anosmia	0.27 (0.13 to 0.57)	0.001

Multivariable Regression Model for Diarrhea	Odds Ratio (95% CI)	*P* Value	Multivariable Regression Model for ICU Admission	Odds Ratio (95% CI)	*P* Value
Age	0.99 (0.97 to 1.00)	0.22	Age	1.04 (1.02 to 1.06)	<0.001
Gender	0.67 (0.36 to 1.27)	0.217	Gender	1.94 (1.12 to 3.36)	0.018
Obesity	0.47 (0.20 to 1.23)	0.088	Obesity	1.67 (0.90 to 3.13)	0.104
ACE-I or ARB	2.05 (1.07 to 3.95)	0.031	ACE-I= or ARB	0.54 (0.30 to 0.97)	0.041
Immunosuppressants	1.94 (0.86 to 4.36)	0.109	Immunosuppressants	2.60 (1.20 to 5.63)	0.016
Tobacco Use	3.47 (0.99 to 12.08)	0.051	≥1 GI Symptom	0.99 (0.56 to 1.74)	0.974
Alcohol Use	1.40 (0.36 to 5.52)	0.627	Myalgia	0.46 (0.24 to 0.89)	0.021
Fever	1.53 (1.04 to 4.32)	0.241	Fatigue	0.90 (0.52 to 0.16)	0.715
Ageusia or Anosmia	2.12 (1.04 to 4.32)	0.039	Ageusia or Anosmia	0.48 (0.19 to 1.23)	0.125
